# Diagnostic CT–guided online adaptive radiotherapy for locally advanced colon cancer: a prospective implementation and feasibility study

**DOI:** 10.1016/j.ctro.2026.101162

**Published:** 2026-04-16

**Authors:** Jun Zhao, Hui Zhang, Lei Yu, Yiwen Hu, Yanju Yang, Sixue Dong, Jing Mi, Yingtao Fang, Jian Qiao, Fan Xia, Weigang Hu, Zhen Zhang

**Affiliations:** a Department of Radiation Oncology, Fudan University Shanghai Cancer Center, Shanghai 200032, China; b Department of Oncology, Shanghai Medical College, Fudan University, Shanghai 200032, China; c Shanghai Clinical Research Center for Radiation Oncology, China; d Shanghai Key Laboratory of Radiation Oncology, Shanghai 200032, China

**Keywords:** CT-guided adaptive radiotherapy, Colon cancer, Online ART, Workflow efficiency, Dosimetric accuracy

## Abstract

**Purpose:**

This study aimed to systematically evaluate the geometric, dosimetric, and workflow characteristics of diagnostic CT-guided adaptive radiotherapy for locally advanced colon cancer (LACC), and to assess its feasibility and efficiency in routine clinical practice.

**Methods and materials:**

Forty patients undergoing short-course radiotherapy on a CT-linac within the TORCH-C protocol were included. ART was triggered when the predefined clinical judgment deemed target coverage on the anatomy of the day inadequate. Interfractional target variation was quantified using Dice similarity coefficient (DSC), Hausdorff distance (HD95), and average symmetric surface distance (ASSD). Dosimetric impact was assessed by recalculating the initial reference plan on the daily CT and comparing it with the adapted plan. In-vivo three-dimensional (3D) gamma verification was performed to evaluate the delivery accuracy. Workflow time and acute toxicity (CTCAE v5.0) were recorded for efficiency and safety analysis.

**Results:**

Among 200 fractions, 48 (24%) required adaptation. Adaptation was highly segment-dependent, occurring in 51.43% of transverse, 40% of ascending, 16% of descending, and 12% of sigmoid colon fractions. Interfractional instability was segment-dependent, with the largest geometric deviation in the transverse colon (median DSC = 0.70; HD95 = 37.9  mm; ASSD = 6.5  mm). Online ART significantly improved target coverage (PTV V100: 67.91% vs 98.25%) while reducing bowel exposure in the low-to-intermediate dose range. The median in-vivo gamma passing rates exceeded 95%. The median ART session duration was 24.6  min. Acute toxicities were mild (3 Grade 3, 1 Grade 2, 14 Grade 1, 22 Grade 0).

**Conclusions:**

Diagnostic CT-guided online ART for LACC is feasible, efficient, and safe within routine clinical throughput, and adaptation occurred in a substantial proportion of fractions, indicating that CT-guided ART can be selectively implemented to restore target coverage and reduce bowel dose in routine colon cancer radiotherapy.

## Highlights

• CT-guided online ART is safe and feasible for LACC .
• ART restores target coverage on the anatomy of the day and reduce dose to normal bowel.
• Online adaptation occurs frequently (24%) and varies by colon segment.
• ART workflow is efficient, with a median treatment time of 24.6  min.

## Introduction

Colorectal cancer is one of the most common malignancies worldwide. Colectomy with en bloc removal of the regional lymph nodes is the main treatment for colon cancer. However, neoadjuvant therapy is increasingly considered for patients with bulky nodal disease or clinical T4b tumors to improve resectability, and the National Comprehensive Cancer Network (NCCN) guidelines recommend neoadjuvant radiation for selected non-metastatic, medically inoperable, or technically unresectable T4 lesions [1]. For locally advanced colon cancer (LACC), however, clinical trials evaluating neoadjuvant chemotherapy alone, including PRODIGE-22, FOxTROT, and NEOSCOPE, have failed to demonstrate substantial improvements in pathological or survival outcomes, highlighting the urgent need to explore novel multimodal strategies. Recent studies in proficient mismatch repair or microsatellite stability (pMMR/MSS) rectal cancer have shown that the combination of radiotherapy, chemotherapy, and PD-1 blockade can induce profound tumor regression [2], [3], inspiring the extension of this approach to LACC [4]. Supporting this shift, neoadjuvant chemoradiotherapy (NACRT) has been reported to significantly increase the R0 resection rate (80% vs. 20%) and improve both progression-free and overall survival compared to neoadjuvant chemotherapy alone [5].

Despite these promising developments, the colon remains one of the most challenging sites for radiotherapy due to large daily variations in position, peristaltic motion, and close proximity to radiosensitive organs such as the small bowel. The position deviation some times even exceed 10 cm [6]. These anatomical and motion-related uncertainties hinder the safe delivery of conformal radiation and have historically limited the role of radiotherapy in LACC management.

Online adaptive radiotherapy (ART) offers a potential solution by enabling daily plan modification to account for anatomical changes. Several online ART platforms have been introduced into clinical practice for various cancer sites [7], [8], [9], [10], [11], [12], [13], [14], [15], [16], including cone beam computed tomography (CBCT) guided systems and magnetic resonance imaging (MRI) guided systems. These technologies have demonstrated both feasibility and clinical value by improving dosimetric accuracy and reducing marginal misses associated with interfraction motion [17], [18], [19], [20], [21]. However, CBCT is limited by poor soft-tissue contrast and often requires fiducials, while MRI-guided ART, though providing excellent visualization, is constrained by prolonged session times (>40 minutes) and limited accessibility. In contrast, diagnostic CT-integrated linear accelerators (CT-linacs) [22] combine high-quality volumetric imaging with streamlined online adaptation workflows, offering a practical platform for widespread clinical implementation.

Within this context, the TORCH-C regimen, which is a multimodality protocol combining short-course radiotherapy (25 Gy in 5 fractions) with CAPOX chemotherapy and PD-1 blockade [4], provides a unique opportunity to evaluate online ART for highly mobile abdominal targets. Integrating CT-guided online ART into this regimen could improve dosimetric accuracy, maintain workflow efficiency, and enhance treatment safety.

The present prospective study was designed to evaluate the implementation feasibility, dosimetric effects, and safety of diagnostic CT-guided online ART for patients with LACC treated within the TORCH-C protocol. Specifically, we aimed to (1) characterize the observed frequency and segment-specific distribution of ART triggered by target deformation, (2) quantify geometric and dosimetric differences between reference and adaptive plans, and (3) evaluate delivery accuracy through in-vivo verification, workflow duration, and acute toxicity associated with this workflow. By establishing practical benchmarks for CT-guided adaptive workflows, this study aims to provide evidence supporting the broader integration of CT-guided ART into routine clinical practice for colon cancer radiotherapy.

## Methods and materials

### Patients

From May 2023 to December 2024, 40 patients with locally advanced colon cancer (LACC) characterized by pMMR/MSS and presenting with cT4-stage bulky tumors or bulky lymph nodes were prospectively enrolled. All patients received the TORCH-C regimen. The present analysis focuses exclusively on the radiotherapy component. The study was approved by the institutional ethics committee.

### Initial reference treatment planning

Initial reference treatment plans were generated based on each patient’s anatomy as visualized on the planning CT. The planning CT was acquired on a simulation CT scanner (Siemens SOMATOM Definition AS) in helical scan mode, with a tube voltage of 120 kV, 300 mAs, a slice thickness of 3 mm, and a field of view of 50 cm. Patients were positioned prone using immobilization devices based on tumor location. For tumors located in the pelvis, both arms were placed on the chest and supported with a headrest and footrest. For tumors located in the abdomen, patients were positioned on a multifunctional board with both arms raised. The gross tumor volume (GTV) was delineated on planning CT with the assistance of MRI, contrast enhanced CT or PET-CT, depending on availability. The GTV was delineated to encompass the primary tumor lesions, adjacent tissues and organs demonstrating tumor infiltration, as well as lymph nodes identified as positive on MRI or CT imaging. Notably, prophylactic irradiation of regional lymphatic drainage areas was not performed. In some cases surgical clips were placed to demarcate the inferior tumor border. The planning target volume (PTV) was generated by applying a 5–10 mm isotropic margin to the GTV. The specific margin size was individualized according to tumor location and anticipated motion related to adjacent anatomical structures, with daily CT guidance used for target position verification. Organs at risk (OARs), including the small bowel, uninvolved colon, bladder, duodenum, femoral head, liver, kidneys and spinal cord, were delineated using the auto-delineation model on the simulation CT according to tumor location. Automatic OAR segmentation was performed using RTP-Net [23], a deep learning–based framework developed for rapid and accurate whole-body organ-at-risk delineation in radiotherapy. RTP-Net has been validated for the segmentation of 65 OARs across the head, thoracic, abdominal, and pelvic regions, including anatomically complex and deformable structures such as the small bowel and colon [23]. All automatically generated contours were reviewed and adjusted if necessary by experienced clinicians before treatment planning.

The prescription dose was 25 Gy in 5 fractions, prescribed to the PTV, with the planning objective of ensuring that at least 95% of the PTV received the prescribed dose. No fixed dose limits were imposed on the OARs; the planning principle was to minimize radiation exposure to all uninvolved structures as much as reasonably achievable. All treatment plans were generated in the uTPS (version R001, United Imaging Healthcare, Shanghai, China) using the VMAT technique with 6-MV photon beams. Dose calculation was performed using a convolution-based algorithm with a calculation grid size of 2.5 mm. The plans were delivered using two partial arcs, with arc angles individualized according to tumor location.

### CT-guided online ART workflow

The CT-guided ART technique was implemented using a CT-linac system that integrates a 16-slice helical CT scanner (70-cm bore) with a C-arm linear accelerator (uRT-linac 506c, United Imaging Healthcare, Shanghai, China) [22]. Each treatment fraction began with a diagnostic-quality pre-treatment verification CT acquired on the CT-linac system in helical scan mode with a tube voltage of 120 kVp, 180 mAs, a slice thickness of 3 mm, and a field of view of 50 cm. The verification image was automatically rigidly registered to the reference planning CT based on bony anatomical structures, including the vertebral bodies and pelvis. After alignment, the physician visually evaluated whether the target remained adequately encompassed by the reference PTV. If coverage was deemed satisfactory and no major anatomical changes were observed, the fraction was delivered using the reference plan. If the target exhibited significant deformation or displacement resulting in partial PTV under coverage, an online adaptive workflow was initiated.

Upon adaptation, the GTV rigidly propagated from the reference plan was manually modified by the physician on the daily CT image, with the reference diagnostic images (contrast-enhanced CT, MRI, and/or PET-CT) displayed on a secondary workstation to assist target delineation. A new PTV was then generated by applying a 5–10 mm isotropic margin. OARs were automatically delineated by the TPS [23] and edited as necessary. An adaptive treatment plan was optimized using the same dose prescription, beam configuration and beam delivery technique as the reference plan. Plan approval required visual inspection and target coverage confirmation by the treating physician and physicist. Once approved, a verification CT was acquired to confirm alignment before delivery. If the target remained within the adapted PTV and patient setup was satisfactory, treatment proceeded; if not, the fraction was postponed at the discretion of the clinical team.

**Fig. 1:**
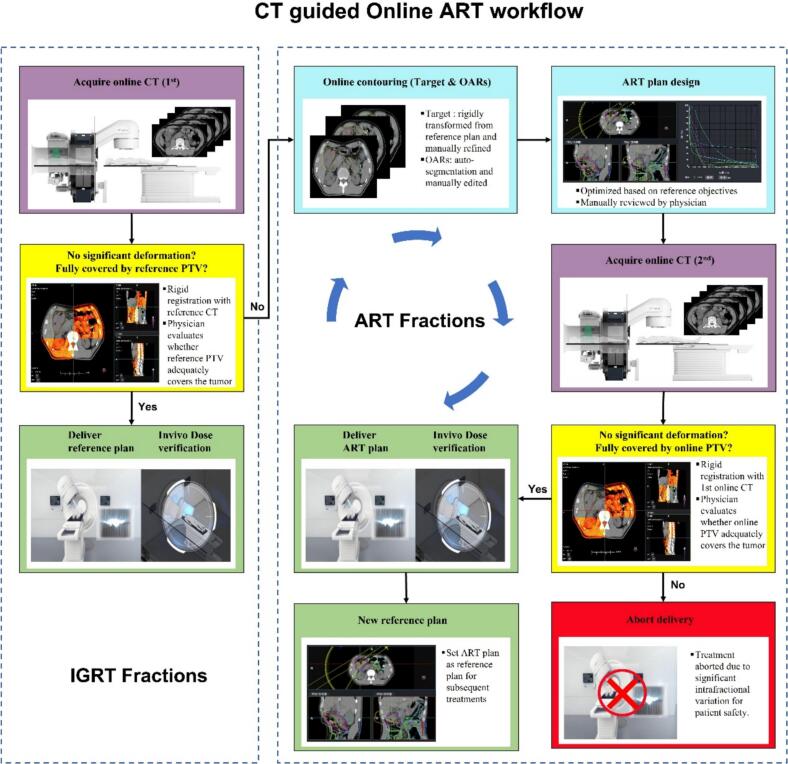
Workflow of diagnostic computed tomography–guided online adaptive radiotherapy (CTgART) for locally advanced colon cancer. Image-guided radiotherapy (IGRT) fractions refer to treatment fractions delivered without online adaptation. Adaptive radiotherapy (ART) fractions refer to treatment fractions in which the target volumes and organs at risk (OARs) are re-contoured based on the daily CT image, and the treatment plan is subsequently re-optimized prior to delivery.

During beam delivery, the CT-linac performed real-time in vivo transit dose verification, providing immediate feedback on treatment accuracy. Following treatment completion, the same transit dose data were reconstructed into three-dimensional (3D) dose distributions on the daily CT anatomy for post-treatment verification. Fig. 1 illustrates the complete workflow of CT-guided online ART, outlining each key procedural step from image acquisition to treatment delivery.

### Geometric and dosimetric evaluation

To quantify interfractional anatomical variations, geometric comparisons were performed between the initial reference and adapted datasets. The Dice similarity coefficient (DSC), 95th percentile Hausdorff distance (HD95), and average surface-to-surface distance (ASSD) were used to evaluate volumetric overlap, extreme boundary deviation, and mean surface discrepancy, respectively, for the GTV and PTV in each ART fraction. Geometric metrics and adaptation frequency was analyzed overall and stratified by colon segment (ascending, transverse, descending, sigmoid) to evaluate regional differences in stability.

To evaluate the dosimetric benefit of CT-guided online ART, the initial reference plan (PI) was rigidly mapped and recalculated on the daily CT to represent the delivered dose if no adaptation had been performed. The corresponding adaptive plan (PA), generated on the same daily CT, was then used for comparison.

For the GTV and PTV, V100 and D95 were evaluated. For the small bowel, V11Gy, V25Gy, D0.1cc, and D5cc were analyzed. For the uninvolved colon, V18.5Gy and V23.25Gy were evaluated. V11Gy for small bowel and V18.5Gy/V23.25Gy for colon, which are related to gastrointestinal toxicities, were derived through EQD2 conversion (assuming an α/β ratio of 3.9 Gy) based on previous findings [24], [25]. Statistical comparisons between PI and PA were performed using the Wilcoxon signed-rank test to determine the significance of any observed differences.

### Workflow efficiency and in-vivo treatment verification

Workflow timestamps recorded by the system were used to quantify the time required for each procedural step, including image acquisition, contour editing, plan optimization, and plan delivery. These data were used to assess overall treatment efficiency and identify time-consuming components of the adaptive workflow.

In vivo treatment verification was performed by comparing reconstructed doses from EPID measurements with corresponding TPS-calculated doses. Gamma analysis was used with a 3%/2 mm gamma criterion and a 10% dose threshold. The results provided confirmation of treatment accuracy and intrafractional stability during beam delivery.

### Toxicity assessment

Adverse events of radiotherapy were monitored and documented from the start of radiotherapy to before the immunochemotherapy and were evaluated according to the Common Terminology Criteria for Adverse Events (CTCAE, version 5.0).

## Results

### Patient characteristics and adaptation frequency

A total of 40 patients with LACC underwent CT-guided short-course radiotherapy within the TORCH-C protocol, accounting for 200 delivered fractions. According to the predefined clinical ART workflow, 48 of 200 fractions (24.0%) required online adaptation due to target deformation compromising PTV coverage, while the remaining 152 fractions were delivered using the reference plan under daily image guided radiotherapy (IGRT) conditions. Nineteen of 40 patients (47.5%) underwent ART for at least one fraction. Thirteen men and six women underwent online adaptation, with a median age of 52 years (range: 18–76 years). Table 1 provides a detailed breakdown of patient demographics, disease stage, ART patient counts, and the proportion of ART fractions per colon segment. The adaptation frequency varied across colon segments and indicate a markedly higher deformation-driven instability in proximal colon regions compared with distal segments.

**Table 1:** Tumor location, patient characteristics, disease stage and ART utilization across colon segments.

Tumor location	No. of patients (male/female)	T3/T4a/T4b staging	No. of ART patients (male/female)	T3/T4a/T4b for ART patients	ART fractions/total fractions
Ascending colon	7(4/3)	0/0/7	6 (4/2)	0/0/6	18/35 (51.43%)
Transverse colon	7 (5/2)	0/1/6	6 (4/2)	0/1/5	14/35 (40.00%)
Descending colon	5(3/2)	0/0/5	1 (1/0)	0/0/1	4/25 (16.00%)
Sigmoid colon	21 (15/6)	2/0/19	6 (4/2)	1/0/5	12/105 (11.43%)

### Interfraction target deviation

**Fig. 2:**
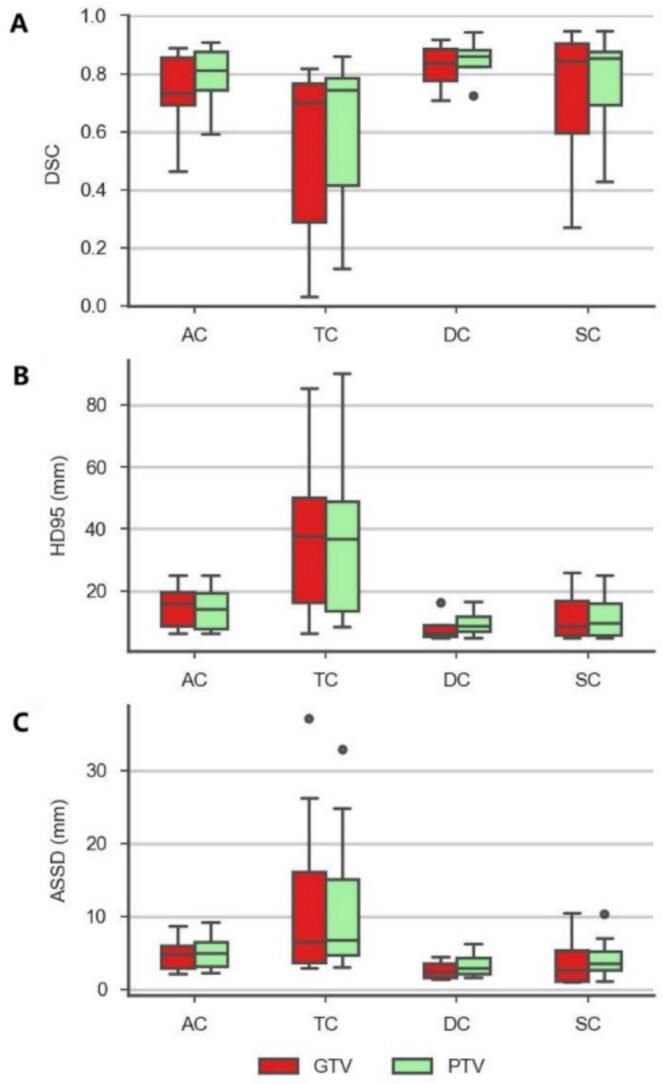
Boxplots of interfractional geometric variation for gross tumor volume (GTV, red) and planning target volume (PTV, green) across colonic sub-sites. Shown are the distributions of (A) Dice similarity coefficient (DSC), (B) 95th-percentile Hausdorff distance (HD95), and (C) average symmetric surface distance (ASSD) for tumors located in the ascending colon (AC), transverse colon (TC), descending colon (DC), and sigmoid colon (SC).

**Fig. 3:**
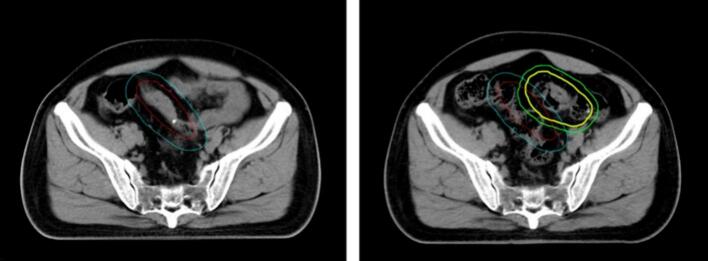
Representative transverse CT slice from the case with the lowest DSC for the target volume. The left panel shows the reference CT, and the right panel shows the corresponding online CT of the last fraction. Red and blue contours represent the GTV and PTV on the reference CT, respectively, while yellow and green contours represent the GTV and PTV on the online CT.

Substantial interfraction geometric variability was observed, with marked differences across colon segments (Table 2 and Fig. 2). Fig. 3 illustrates a representative case with the lowest DSC between the reference and online target volumes. When pooled across all ART fractions, the median DSC, HD95, and ASSD for GTV were 0.75, 15.00 mm, and 4.63 mm, respectively. Transverse-colon tumors exhibited the greatest interfraction displacement and shape variability, with the lowest DSC and highest HD95/ASSD values among all sub-sites. In contrast, descending colon lesions showed comparatively stable positioning with minimal deformation. Sigmoid colon lesions likewise exhibited relatively stable positioning, although with greater shape deformation compared with the descending colon. PTV metrics demonstrated the same regional trend. These data support that the magnitude of anatomical variation was site-dependent and greatest in the proximal colon.

**Table 2:** Geometric metrics of interfractional variation of GTV and PTV for ART fractions.

	GTV					PTV				
tumor sites (ratio of ART fractions)	Reference volume (cc)	ART volume (cc)	DSC	HD95(mm)	ASSD (mm)	Reference volume (cc)	ART volume (cc)	DSC	HD95(mm)	ASSD (mm)
Ascending colon (18/35)	157 (68–474)	219 (62–462)	0.73 (0.69–0.86)	15.94 (8.90–19.76)	4.91 (2.97–5.97)	333 (153–693)	420 (154–621)	0.81 (0.74–0.88)	14.34 (7.97–19.33)	5.05 (3.15–6.48)
Transverse colon (14/35)	110 (55–312)	99 (48–247)	0.70 (0.29–0.77)	37.85 (16.42–50.00)	6.47 (3.76–16.08)	245 (167–494)	221 (114–443)	0.74 (0.42–0.79)	36.82 (13.66–48.75)	6.78 (4.73–15.12)
Descending colon (4/25)	96 (N/A)	113 (N/A)	0.83 (0.77–0.89)	6.28 (5.53–9.18)	2.42 (1.60–3.52)	196 (N/A)	228 (N/A)	0.86 (0.83–0.88)	8.82 (6.97–11.67)	2.97 (2.15–4.28)
Sigmoid colon (12/105)	151 (55–257)	165 (42–254)	0.85 (0.60–0.90)	8.89 (5.65–16.79)	2.65 (1.14–5.32)	296 (114– 495)	327 (96–445)	0.85 (0.69–0.87)	9.74 (5.85–10.05)	3.52 (2.65–5.27)
Colon (all together) (48/200)	122 (55–474)	123 (42–462)	0.75 (0.65–0.87)	15.00 (7.58–22.60)	4.63 (2.71–6.75)	243 (114–693)	267 (96–621)	0.79 (0.70–0.86)	14.21 (7.7–22.7)	5.03 (3.03–6.79)

Reference and ART volumes represent the structure volumes delineated on the reference planning CT and the corresponding daily online CT, respectively. Volumetric data are presented as Median (Range: min–max). For ART volumes, a two-step statistical approach was employed to ensure equal weighting per subject by first averaging volumes across adapted fractions at the patient level before deriving group-level statistics. For the Descending Colon (DC) subgroup (1patient), the range is marked as N/A (not applicable). Geometric similarity metrics, including Dice similarity coefficient (DSC), 95th percentile Hausdorff distance (HD95), and average symmetric surface distance (ASSD), are presented as median with interquartile range (IQR) across ART fractions.

### Dose–volume effects of adaptive replanning

**Fig. 4:**
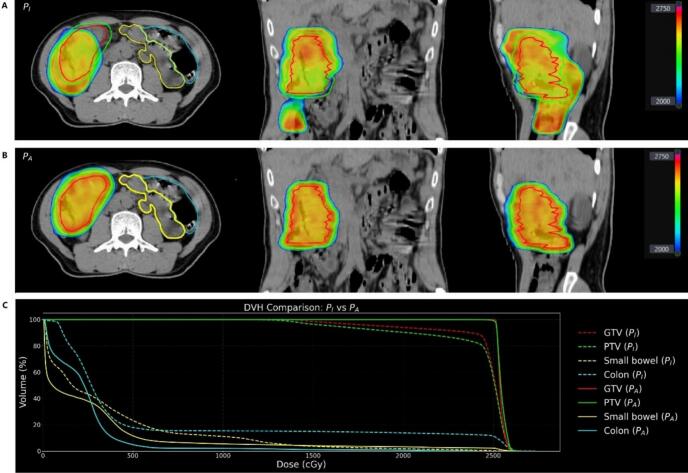
Dose comparison between the initial reference plan and the adapted plan on the daily CT image. (A) Initial reference plan (PI) recalculated on the daily CT. (B) Adapted plan (PA) generated after re-contouring and re-optimization. (C) Dose–volume histogram (DVH) comparing the two plans, with solid lines representing the adapted plan and dashed lines representing the initial plan. Structures are color-coded as follows: gross tumor volume (GTV, red), planning target volume (PTV, green), small bowel (yellow), and uninvolved colon (blue).

Adaptive replanning consistently restored target dose coverage and improved conformity on the daily anatomy. As illustrated in Fig.4, the adapted plan achieved full prescription coverage while maintaining or reducing OAR dose compared with the recalculated initial plan, with the caudal high-dose region in the initial plan attributable to anatomical changes and target deformation. Aggregated dosimetric results for ART fractions are summarized in Table 3. For the PTV, mean V100 improved from 67.91% (PI) to 98.25% (PA), and D95 increased from 16.96 Gy to 25.22 Gy. Similar gains were observed for GTV coverage. Regarding OARs, ART significantly reduced low to intermediate dose exposure to uninvolved bowel, such as small-bowel V11Gy and uninvolved colon V18.5Gy and V23.25Gy. High-dose bowel metrics (D0.1cc, D5cc) remained comparable between PI and PA. Together, these findings indicate that ART not only corrected geometric miss but also translated into meaningful dosimetric benefit without increasing OAR high-dose burden.

**Table 3:** Target coverage and dosimetic parameters for organ at risks.

		P_I_ mean (SD)	P_I_ median (range)	P_A_ mean (SD)	P_A_ median (range)	*p*
PTV	V100 (%)	67.91 ± 21.95	72.21(10.11–95.28)	98.25 ± 0.96	98.06 (96.00–99.76)	0.00
	D95 (Gy)	16.96 ± 7.76	18.94 (0.66–26.80)	25.22 ± 0.26	25.17 (25.06–26.56)	0.00

GTV	V100 (%)	78.98 ± 23.18	86.99 (9.43- 99.88)	99.99 ± 0.06	100.00 (99.62–100.00)	0.00
	D95 (Gy)	20.38 ± 7.74	24.30 (0.82–26.70)	25.33 ±0.24	25.28 (25.11–26.43)	0.00

Small bowel	V11Gy (cc)	77.34 ± 61.59	66.95 (0.97–236.09)	69.66 ± 54.04	61.95 (1.48–205.41)	0.02
	V25Gy (cc)	11.35 ± 14.13	6.52 (0.00- 54.56)	8.93 ± 9.38	6.53 (0.00–34.9)	0.74
	D0.1cc	24.69 ± 2.81	25.73 (16.55–27.04)	25.41 ± 1.54	25.79 (15.88–26.59)	0.80
	D5cc	21.44 ± 5.64	25.14 (8.42–26.78)	22.46 ± 4.5	25.18 (9.48- 26.19)	0.11

Colon	V18.5Gy (cc)	30.08 ± 41.95	16.36 (0.00–201.55)	19.69 ± 21.28	13.72 (0.00–105.18)	0.03
	V23.25Gy (cc)	21.24 ± 33.31	10.94 (0.00–158.23)	11.74 ± 15.41	6.45 (0.00–71.81)	0.00

PI: initial reference plan; PA: adaptive plan; OAR: organ at risk. p-values were calculated using the Wilcoxon signed-rank test.

### Workflow efficiency

**Fig. 5:**
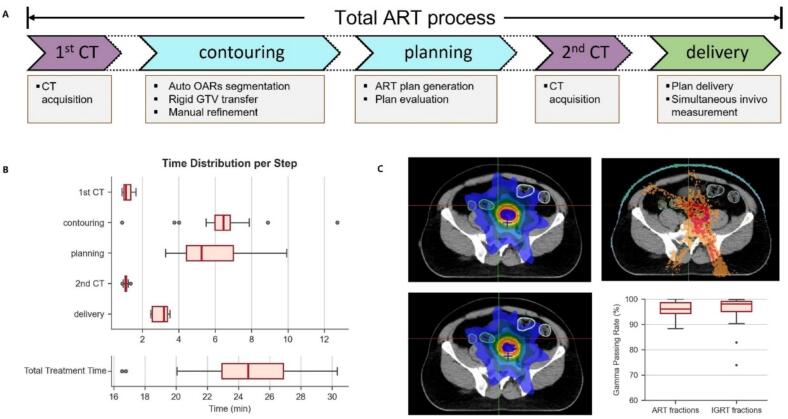
(A) Steps of the adaptive radiotherapy (ART) workflow for which timing data were collected, along with a brief description of each step. Dashed lines indicate additional operations that may occur between the main steps but are not explicitly shown. (B) Timing data for each workflow step, including the total duration of ART delivery. (C) In vivo 3D dose comparison for a representative case, showing planned dose, reconstructed dose, and dose difference, together with boxplots of 3D gamma passing rates for ART and IGRT fractions.

Fig. 5A shows the critical steps in ART workflow. Fig. 5B. shows the timing data for each step, from the initiation of the first CT scan to the completion of beam delivery. The median total online ART session time was 24.61 minutes (range 16.53–30.33). Median duration for key procedural steps was 1.08 min for pre-treatment CT, 6.46 min for contour editing, 5.26 min for re-planning, 1.09 min for verification CT, and 3.17 min for treatment delivery. All ART fractions were completed within a clinically acceptable timeframe. In most cases, the adaptive treatment plan was finalized after a single optimization iteration, while two optimization iterations were required in a subset of cases. In general, a single optimization iteration typically required approximately 4 minutes.

### In-vivo treatment verification

Fig. 5C shows a representative case of in vivo 3D dose comparison, including the planned dose distribution, reconstructed dose from in vivo measurements, and dose difference map. In-vivo 3D dose verification confirmed high delivery accuracy for both ART and IGRT fractions. Median gamma passing rates were 95.6% for ART fractions and 97.9% for IGRT fractions. Only 1 ART fractions and 2 IGRT fractions have a gamma passing rate lower than 90%.

### Acute toxicity outcomes

Treatment was well tolerated. Among the 40 patients, no treatment interruption occurred due to acute toxicity. As summarized in Table 4, treatment-related adverse events were generally mild. The most common reported adverse event was abdominal pain, reported in 8 patients (20%), followed by diarrhea in 6 patients (15%). Other GI toxicities included rectal bleeding in 1 patient and intestinal obstruction in 2 patients. Urinary frequency was reported in 1 patient. Notably, more than half of the cohort (n = 22, 55%) experienced no acute gastrointestinal or urinary toxicities during treatment.

**Table 4:** Acute adverse events of radiotherapy (CTCAE v5.0).

Adverse event	Grade	Frequency (n)
Abdominal pain	Grade 1	7
Abdominal pain	Grade 2	1
Diarrhea	Grade 1	5
Diarrhea	Grade 3	1
Rectal bleeding	Grade 1	1
Intestinal obstruction	Grade 3	2
Urinary frequency	Grade 1	1
No adverse events reported	Grade 0	22

Adverse events were assessed from the start of radiotherapy to before immunochemotherapy according to CTCAE v5.0.

## Discussion

This prospective study demonstrates that diagnostic CT–guided online ART is both feasible and clinically actionable for LACC within the TORCH-C regimen, with measurable gains in target coverage and consistent sparing of surrounding bowel while maintaining a streamlined workflow suitable for routine practice. The need for adaptation was not rare, occurring in one out of four fractions, and was driven entirely by target deformation rather than setup uncertainty. This finding reinforces that immobilization and IGRT alone are insufficient for highly mobile colonic targets.

A key observation was the segment-dependent nature of anatomical instability. Tumors located in the transverse and ascending colon showed the highest ART trigger rate and the largest geometric deviations, consistent with their greater physiological mobility and peritoneal redundancy. In contrast, sigmoid and descending colon tumors were more geometrically stable and less frequently required adaptive intervention. This segmental heterogeneity is important for treatment planning because it suggests that not all LACC need the same adaptive intensity, therefore presenting a potential direction for personalized ART workflows.

From a dosimetric standpoint, ART reliably restored full-prescription target coverage on the anatomy of the day, while simultaneously reducing low and intermediate dose exposure to bowel without increasing high dose burden. This is particularly relevant in the neoadjuvant setting where gastrointestinal toxicity can delay systemic therapy or compromise surgical tolerability. Although the study was not designed to detect clinical endpoints, the directional dosimetric improvements suggest that CT-guided ART may enhance the therapeutic window of short course radiotherapy for mobile abdominal disease.

Unlike CBCT-guided ART platforms, CT-linac systems provided adequate soft-tissue contrast without the need for fiducials and avoided the prolonged session times reported for MRI-guided ART. In this cohort, the median total session duration was 24.6 minutes, which is substantially shorter than the approximately 50 minutes typically reported for MRI-based ART in abdominal and pelvic sites [26], [27], [28], and modestly shorter than the approximately 34 minutes reported for CBCT-based adaptation workflows [29], [30]. Although noticeable variability in replanning time was observed, this primarily reflected case-specific planning complexity, including differences in target volume size, target geometry, and arc configuration, rather than workflow instability. This level of efficiency indicates that CT-guided online ART can be integrated into routine clinical throughput without meaningful disruption. In vivo verification further confirmed acceptable delivery accuracy across both adaptive and non-adaptive fractions, supporting the safety of online replanning under real-world treatment conditions.

Despite these strengths, intrafractional motion remains an unresolved challenge for highly deformable abdominal targets. In two fractions, a second verification CT acquired approximately twelve minutes after the pre-adaptation scan revealed new target deformation sufficient to invalidate the approved adaptive plan, prompting treatment cancellation for patient safety. These events illustrate the intrinsic limitation of single-timepoint adaptation: anatomic conditions can evolve during the adaptation window and compromise plan validity. This observation has direct workflow implications. A verification scan immediately before beam-on may serve as a necessary safeguard in high-mobility sites, and it is notable that such a step is absent from current MRI-based ART workflows such as those implemented on Unity systems. Patient-centered measures, such as dietary guidance to reduce intraluminal gas, may further improve reproducibility.

Future research should move beyond geometric and dosimetric surrogates toward testing ART-triggered strategies in prospective cohorts with predefined clinical endpoints, and to determine whether segment-specific or risk-adapted ART utilization can improve toxicity, surgical readiness, or treatment compliance in mobile abdominal malignancies. This study is limited by the modest number of adapted fractions and the absence of longitudinal outcomes; consequently, it cannot establish clinical benefit or define threshold-based triggers. Furthermore, although volumetric differences between reference and adapted GTVs were generally modest, observer-related variability remains an inherent consideration in online adaptive workflows. The fact that adaptation was performed only for a subset of fractions based on clinical triggers may also introduce selection bias; thus, future multi-observer analyses involving larger patient cohorts and more comprehensive fraction coverage are warranted to further quantify contour reproducibility and its subsequent clinical impact. In addition, the evaluation focused on day-specific geometric and dosimetric effects rather than linking ART use to downstream endpoints such as immunochemotherapy tolerance or operability. Nevertheless, by providing real-world adaptive rates, segment-dependent instability, and verified workflow tolerability, this first prospective evaluation of CT-guided online ART for LACC provides implementation-level benchmarks that can inform the design of outcome-powered trials and guide selective ART deployment in practice.

## Conclusion

This is the first prospective evaluation of diagnostic CT–guided online ART for locally advanced colon cancer. The ART workflow was feasible within routine clinical throughput, consistently restored target coverage on the anatomy of the day, and reduced bowel exposure without increasing toxicity or compromising delivery accuracy. Adaptation was non-rare and strongly segment-dependent, indicating that IGRT alone is insufficient for mobile colonic targets and that ART can be deployed as a rational, trigger-based intervention rather than a universal mandate. These results establish disease-specific implementation benchmarks and provide a practical foundation for future outcome-driven trials of CT-guided ART.

## CRediT authorship contribution statement

**Jun Zhao:** Conceptualization, Data curation, Formal analysis, Visualization, Writing – original draft. **Hui Zhang:** Writing – review & editing. **Lei Yu:** . **Yiwen Hu:** Data curation. **Yanju Yang:** Data curation. **Sixue Dong:** Data curation. **Jing Mi:** Data curation. **Yingtao Fang:** Data curation. **Jian Qiao:** Data curation. **Fan Xia:** Conceptualization, Writing – review & editing. **Weigang Hu:** Conceptualization, Writing – review & editing. **Zhen Zhang:** Conceptualization.

## Funding

This work was supported by the National Key Research and Develop Program of China (2023YFC2413900, 2022YFC2407100, 2022YFC2404603, 2022YFC2407100), the National Natural Science Foundation of China (12375339, 82003229, 12475339) and Shanghai Committee of Science and Technology Fund (24ZR1413300, 25TS1405300).

## Declaration of competing interest

The authors declare that they have no known competing financial interests or personal relationships that could have appeared to influence the work reported in this paper.
